# Clinically guided core biopsy and cutaneous punch biopsy in the evaluation of breast lesions: a necessary test or an obsolete skill?

**DOI:** 10.1007/s11845-022-02937-8

**Published:** 2022-02-08

**Authors:** Aqeel Alameer, Matthew Common, Sami Abd Elwahab, Michael Boland, Michael Allen, Colm Power, Niamh Hambly, Jennifer Kerr, Neasa Ni Mhuircheartaigh, Marie Staunton, Arnold D. K. Hill, Deirdre Duke

**Affiliations:** 1 Department of Surgery, Beaumont Hospital, Dublin, Ireland; 2 Department of Radiology, Beaumont Hospital, Dublin, Ireland; 3 Department of Pathology, Beaumont Hospital, Dublin, Ireland; 4 Department of Radiology, Ninewells Hospital and Medical School, Dundee, UK

**Keywords:** Breast cancer, Breast imaging, Breast lump, Core biopsy, Mammography, Punch biopsy, Ultrasonography

## Abstract

**Objective:**

The vast majority of breast cancers are diagnosed via image-guided procedures yet despite significant advances, imaging does not identify all breast malignancies. Clinically suspicious breast lesions with normal breast imaging remain a cause for concern. The aim of this study is to determine the diagnostic value of clinical core and cutaneous punch biopsies in the diagnosis of breast malignancy in clinically suspicious lesions with normal breast imaging.

**Methods:**

All patients with suspicious clinical breast findings and normal imaging who underwent a clinical core and/or cutaneous punch biopsy from 2012 to 2019 were reviewed retrospectively. Patients with subsequent breast malignant diagnosis were analysed.

**Results:**

A total of 283 biopsies (166 clinical core, 117 cutaneous punch) performed over the 7-year period were included in the analysis. A total of 263/283 (93%) yielded a benign outcome. A total of 2/283 (0.7%) yielded B3 lesions (probably benign). These lesions were benign on final surgical excision. A total of 18/283 (6.3%) yielded a malignant histopathology. Sixteen out of 18 were cutaneous punch biopsies, and 2/18 were clinical core biopsies. A total of 14/18 patients presented with nipple changes, while 4/18 had a palpable area of concern. Histopathological analysis demonstrated Paget’s disease of the nipple in 8/18, invasive carcinoma in 9/18 out of which two represented a recurrence of breast malignancy. Cutaneous squamous cell carcinoma was diagnosed in 1/18.

**Conclusion:**

Clinical core and cutaneous punch biopsies remain a valuable tool in the diagnosis of breast cancer particularly in the management of clinically suspicious radiographically occult malignancies.

## Introduction

Mammography and ultrasonography are the main and most commonly used radiological modalities in the evaluation of breast lesions [1]. The combination of these two modalities significantly reduces the risk of false negative rate for diagnosis of breast malignancy [2]. A negative mammography and ultrasonography does not always exclude breast cancer, and the probability of breast cancer in imaging-occult lesions remains at approximately 2.6–2.7% [2, 3]. Clinically suspicious lesions in the presence of normal breast imaging remain a diagnostic challenge.

The development of image guided biopsy techniques altered the practice of medical specialists including surgeons who in the past relied on clinically guided biopsy or even open surgical biopsies for the evaluation of all breast lesions [4]. Image-guided biopsy, when available and feasible, adds the benefit of targeted biopsy of suspicious lesions, detects multifocal and multicentric lesions, and targets solid suspicious regions in heterogeneous and cystic tumours, thus reducing the risk of false negative biopsies. Blind or clinical core biopsies have a role, especially in the context of clinically suspicious lesions that are imaging occult, when the clinical and radiological examinations are discordant [5].

The purpose of this study is to review the diagnostic value of clinically guided core biopsy and cutaneous punch biopsies in the diagnosis of breast malignancy in palpable clinically suspicious lesions with normal breast imaging.

## Methods

Retrospective data was obtained from Beaumont Hospital Breast Cancer Data Centre for patients who underwent clinically guided core and cutaneous punch biopsies between January 2012 and December 2019. A five-grade coding system was used for clinical assessment. RCR scoring system was used for radiological classification, and B-coding system for histological examination (Table 1). Patients who had a clinically suspicious breast lesion (S4 and S5) and normal breast imaging using mammography and ultrasonography (R1 and R2) were included. Patients who had a malignant histopathological outcome were further analysed.

**Table 1:** Clinical, radiological, and pathological classifications

**Code**	**Clinical classification**	**Code**	**Radiological classification**	**Code**	**Pathological classification**
S1	Normal clinical examination	R1	Normal imaging	B1	Normal
S2	Benign	R2	Benign	B2	Benign
S3	Probably benign	R3	Probably benign	B3	Probably benign
S4	Suspicious	R4	Suspicious	B4	Suspicious
S5	Malignant	R5	Malignant	B5	Malignant

Patients who had image-guided biopsy in addition to clinically guided biopsy and/ or cutaneous punch biopsy were excluded. Patients who had abnormal imaging on mammography and ultrasonography were also excluded.

## Results

There were a total of 304 clinically guided and cutaneous punch biopsies performed in Beaumont hospital breast unit in the period from January 2012 to December 2019. Six patients were excluded as they also had an imaged-guided biopsy. Fifteen patients were excluded as they had abnormal imaging, but an image-guided core biopsy was not feasible. The remaining 283 patients presented with a clinically suspicious breast lesion (S4 or S5) with normal mammography and ultrasonography (R1 or R2). In total, 166 were clinical core biopsies and 117 were cutaneous punch biopsies.

The mean age of patients at presentation was 59 years (range 19–96 years).

In total, 263 (93%) biopsies yielded a benign diagnosis (B1 or B2). Two biopsies (0.7%) resulted in a B3 diagnosis (uncertain/probably benign) which were subsequently confirmed to be benign on final surgical excision.

A total of 18 (6.3%) biopsies in 17 patients resulted in a malignant diagnosis. Of the 18 biopsies with malignant pathology, 16 were cutaneous punch biopsies, while two were clinical core biopsies. Fourteen of these patients presented with nipple areolar complex changes, while four patients presented with palpable areas of concern.

Of the 18 biopsies with malignant pathology, eight were consistent with Paget’s disease of the nipple, nine demonstrated invasive carcinoma, of which two represented a recurrence of breast malignancy and one patient was diagnosed with a cutaneous squamous cell carcinoma (Table 2).

**Table 2:** Histopathology results for patients with malignant biopsy outcomes

**Histopathological diagnosis**	***N***
Paget’s disease of the nipple	8
Invasive carcinoma	9
Cutaneous squamous cell carcinoma	1

## Discussion

The introduction of biopsy using image guidance has significantly increased the rate of detection of breast malignancy with a significant reduction in the number of diagnostic open surgical procedures which often resulted in psychological distress, considerable cost, and scarring of the breast [6]. Unfortunately, a normal mammogram and breast ultrasound does not exclude breast malignancy in all patients particularly in the presence of a clinically suspicious palpable lesion. Therefore, clinically suspicious breast lesions with discordant clinical and radiological findings need to be evaluated with a free-hand or clinically non-image guided biopsy.

The 2015 National Cancer Control Programme (NCCP) guidelines recommend that a clinically suspicious breast lesion (S4, S5) with normal imaging features on mammography and ultrasonography should undergo a clinically guided core biopsy [7]. Our study supports this recommendation particularly in the evaluation of suspicious findings involving the nipple areolar complex. Paget’s disease of the nipple was diagnosed in eight of our patients.

The use and benefit of alternative imaging modalities such as breast MRI to further evaluate suspicious clinical findings with normal mammography and breast ultrasound was not explored in our study. Breast MRI is particularly useful in the assessment of focal suspicious palpable areas of concern [8].

Our study represents a continuation of the efforts to establish a consensus in the management of clinically suspicious lesions with normal imaging. True imaging-occult lesions were quite rare in our patient’s cohort. If one excludes patients with abnormalities of the nipple areolar complex, only 2 of 166 (1.2%) clinically guided core biopsies yielded a malignant diagnosis after normal mammography and ultrasonography which is similar to previous studies [9].

Patients with clinical and radiological discordance are a diagnostic challenge. These patients must be reviewed by the most senior clinician and discussed individually with senior radiologist. Alternatives to clinical core biopsy, such as use of breast MRI, short interval clinical, and/or radiological follow-up, should be explored and discussed. This will ensure that unnecessary clinical core biopsies and their associated negative psychological impact can be avoided while ensuring that imaging occult cancers are diagnosed [10].

## Conclusion

Clinical core and cutaneous punch biopsies remain a valuable tool in the diagnosis of breast malignancy in patients who have clinically suspicious lesions in the presence of normal breast imaging. This is particularly true when the lesion involves the nipple areolar complex. It is therefore imperative that breast clinicians retain these skills.
